# Enhancing the use of exposure science across EU chemical policies as part of the European Exposure Science Strategy 2020–2030

**DOI:** 10.1038/s41370-021-00388-4

**Published:** 2021-10-25

**Authors:** Yuri Bruinen de Bruin, Antonio Franco, Andreas Ahrens, Alick Morris, Hans Verhagen, Stylianos Kephalopoulos, Valeria Dulio, Jaroslav Slobodnik, Dick T.H.M. Sijm, Theo Vermeire, Takaaki Ito, Koki Takaki, Jonathas De Mello, Jos Bessems, Maryam Zare Jeddi, Celia Tanarro Gozalo, Kevin Pollard, Josephine McCourt, Peter Fantke

**Affiliations:** 1 European Commission, Joint Research Centre, Directorate for Space, Security and Migration, Geel, Belgium; 2 European Commission, Joint Research Centre, Directorate on Health, Consumer and Reference Materials, Ispra, Italy; 3 European Chemicals Agency, Helsinki, Finland; 4 European Commission, Directorate General Employment, Luxembourg, Luxembourg; 5 National Food Institute, Technical University of Denmark, Kgs. Lyngby, Denmark; 6 University of Ulster, Coleraine, Northern Ireland; 7 INERIS – National Institute for Environment and Industrial Risks, Verneuil en Halatte, France; 8 Environmental Institute, Koš, Slovakia; 9 Dutch Food and Consumer Product Safety Authority, Utrecht, The Netherlands; 10 University College Venlo, Campus Venlo, Maastricht University, Maastricht, The Netherlands; 11 RIVM – National Institute for Public Health and the Environment, Bilthoven, The Netherlands; 12 Organisation for Economic Co-operation and Development, Paris, France; 13 United Nations Environment Programme, Paris, France; 14 Flemish Institute for Technological Research, Mol, Belgium; 15 Quantitative Sustainability Assessment, Department of Technology, Management and Economics, Technical University of Denmark, Kgs. Lyngby, Denmark; 16 Present Address: European Chemical Industry Council (Cefic), Brussels, Belgium

**Keywords:** Exposure assessment; EU Green Deal; Chemical safety; Chemical security; Environmental sustainability; ISES Europe

## Abstract

**Background:**

A scientific framework on exposure science will boost the multiuse of exposure knowledge across EU chemicals-related policies and improve risk assessment, risk management and communication across EU safety, security and sustainability domains.

**Objective:**

To stimulate public and private actors to align and strengthen the cross-policy adoption of exposure assessment data, methods and tools across EU legislation.

**Methods:**

By mapping and analysing the EU regulatory landscape making use of exposure information, policy and research challenges and key areas of action are identified and translated into opportunities enhancing policy and scientific efficiency.

**Results:**

Identified key areas of actions are to develop a common scientific exposure assessment framework, supported by baseline acceptance criteria and a shared knowledge base enhancing exchangeability and acceptability of exposure knowledge within and across EU chemicals-related policies. Furthermore, such framework will improve communication and management across EU chemical safety, security and sustainability policies comprising sourcing, manufacturing and global trade of goods and waste management. In support of building such a common framework and its effective use in policy and industry, exposure science innovation needs to be better embedded along the whole policymaking cycle, and be integrated into companies’ safety and sustainability management systems. This will help to systemically improve regulatory risk management practices.

**Significance:**

This paper constitutes an important step towards the implementation of the EU Green Deal and its underlying policy strategies, such as the Chemicals Strategy for Sustainability.

## Introduction

Since the inception of the European Union (EU) chemical legislation in the 1960s, the contribution of exposure science to EU legislation has evolved from providing ex-post evidence to enabling prospective identification and control of chemical exposure and risks at workplaces, private homes, in food, human bodies and in environmental compartments. In addition to EU legislation, the role of exposure science is at present also recognised by international law (e.g., Rotterdam, Basel, Stockholm, Minamata, and the Chemical Weapons Conventions) and global policy initiatives focusing on evaluating the safety, security and sustainability of chemicals (e.g., Strategic Approach to International Chemicals Management (SAICM), UN Sustainable Development Goals (SDGs) [1–3], World Business Council for Sustainable Development [4]), as well as by international organisations, such as the Organisation for Economic Co-operation and Development (OECD) programme on chemical safety and biosafety, the International Programme on Chemical Safety of the World Health Organization (WHO) and the UN ILO’s Inter-Organization Programme for the Sound Management of Chemicals.

Across international legislation, exposure science is applied to protect against adverse health effects in humans and the environment from intended and unintended exposures covering the domains of health and safety, security and sustainability [1, 2, 5–10]. As such, the meaning of risk within the scope of this paper is the function of the probability of an adverse health effect due to exposure and the severity of that effect, consequential to a hazard [11]. Specifically, within chemical legislation, exposure assessment together with hazard identification and characterisation are the two fundamental regulatory pillars to inform risk assessment of chemicals.

The current EU chemical’s legislative landscape targeting protection of human and environmental health comprises numerous legislations, each focussing on a specific domain or protection target comprising health, safety, security and sustainability. Thereby, chemical safety refers to measures and conditions to prevent adverse effects via releases of chemicals from products and processes. Chemical security, in contrast, refers to measures to prevent deliberate releases of chemicals with the goal to cause harm to humans, the environment and/or assets, and to mitigate related impacts [12]. Sustainability is a concept in that economy operates within the ecologically planetary limits and that seeks safe and environmentally benign solution [13]. Regarding chemicals, sustainability refers to manufacturing and uses of chemicals (as such or in materials and articles), including reuse and recycling. In this context chemistry strives towards resource efficiency, carbon-neutrality as well as a non-toxic environment. The complexity of the regulatory landscape is the result of policy-specific needs with at times similar protection targets, but different starting points and focus leading towards disparate efforts to address these needs. Regulatory connections are a key component, with provisions and decisions under one piece of legislation impacting other policy areas [2]. The present paper focuses on the specific requirements and actions relevant to materialise the author’s vision improving the use exposure science in the EU regulatory context. Many of the challenges addressed, however, entail a global dimension and call for solutions to be pursued at global level, for example, exposure related to global souring of raw materials for the European market (including mining), import of hazardous substances in articles to Europe and to the export of waste streams from Europe to the parts of the world.

With respect to hazard identification and communication, common global horizontal frameworks have emerged, such as the UN Globally Harmonised System of Classification and Labelling of Chemical substances and mixtures [14–16], implemented in the EU under the Classification, Labelling and Packaging (CLP) Regulation ((EC) No 1272/2008) [17]. In addition, harmonised test guidelines and templates for toxicity, physico-chemical and fate and behaviour properties have been developed at OECD level, and form the basis for consistent exposure and hazard assessment. With the International Uniform Chemical Information Database (IUCLID), an internationally agreed data exchange format has been developed; however, mostly focussing on hazard data.

Achieving the objectives of the EU Green Deal and related policy strategies requiring the EU to become climate neutral, waste-free and non-toxic by 2050, relies on the excellence of exposure science applied within current and future regulatory domains [6, 9, 18, 19]. At the time of writing of this paper, also other geographical areas around the globe adopted or are in the phase of adopting sustainability targets (e.g., China, Korea, US) leading to the recent EU announcement to set up a Global Green Deal [20]. For the pillar of risk assessment, including assessment of uses, mass-flows and exposure data, no such horizontal framework is currently available, leading to diverging assessment and implementation concepts across legislations, thus hampering a harmonised approach to science-based risk management [21]. This includes, for example, differences in taxonomies and regulatory requirements concerning exposure and risk-endpoints, methods of data production (e.g., monitoring), collection (e.g., data repositories) and processing (e.g., mathematical models) [22]. In addition, the currently used risk assessment frameworks usually do not consider the global dimension of exposure pathways and associated risks throughout the life-cycle of chemicals [3]. This includes resource extraction (e.g., mining and ore processing), chemical synthesis and manufacturing, trade and transportation, use and end-of-life for chemicals and related products inside and outside the EU [12, 23]. Moreover, circularity processes (in particular recycle, repurpose, remanufacture, refurbish, repair, reuse, reduce, refuse) (UNEP, [24]) frequently lack the exposure dimension to assess gains and shortcomings associated with a shift towards circular economy. Pursuing the commitments outlined in the European Commission’s recent Chemicals Strategy for Sustainability [7] and related policy initiatives (e.g., circular economy [25], zero-pollution ambition [8]) requires a harmonised, science-based framework for exposure assessment that is able to deal with global flows of materials and assess the impact of related hazardous chemicals, contained and released along material life-cycles.

At the EU level, initial efforts have focused on harmonising exposure assessment methods and tools within individual policy domains falling under the remit of specific European Agencies. This includes, for example, the European Food Safety Authority (EFSA), which developed harmonised models and tools related to food safety, animal, plant and ecological health [26, 27], consumer safety, with the development of the ConsExpo tool by the Dutch National Institute for Public Health and the Environment (RIVM) in international collaboration with the counterpart institutes ANSES (France), BfR (Germany), FOPH (Switzerland) and Health Canada (e.g., [28]), and the European Union System for the Evaluation of Substances (EUSES) for the assessment of environmental exposure under REACH and the Biocidal Products Directive (e.g., [29]). However, despite several efforts to improve processes and information quality [30, 31], a common EU scientific framework on exposure assessment is still lacking [32, 33].

The European Commission has recently completed a series of policy evaluations, including the REACH review [9], the fitness checks of chemical legislation (excluding REACH) [34], the Water legislation [35] and the General Food Law 178/2002. Following the revision of the General Food Law, the Transparency Regulation 2019/1381 [36] resulted in increasing the transparency and sustainability of the EU risk assessment in the food chain. These evaluations have revealed some of the gaps and inefficiencies with respect to the generation and use of exposure information. These include the need to enable the use of exposure datasets and exposure modelling tools across policy areas, to consider open data policies and simplification of their use for private actors with limited resources (e.g., small and medium-sized enterprises), to improve the tracking of substances of concern along material and product life-cycles, to assess chemicals with a grouping approach for operational efficiency gains and to address knowledge gaps for long-term, large-scale complex exposure and risk scenarios. Overall, the current regulatory frameworks have been mainly designed to assess and manage risks from single substances within sectorial domains, and typically do not focus on the short- or long-term effects of complex exposures. Scientists have explored approaches and developed guidance to tackle some of these challenges. This includes the consideration of aggregated exposures [37], the development of a generic mixture assessment factor addressing combined risk from multiple chemicals [38–40], consistently integrating different spatial and temporal exposure scales and settings [41, 42], human biomonitoring (HBM) [27, 43–45] and life-long environmental exposures (the Exposome concept) [46]. However, the inclusion of new approaches and harmonisation across multiple policy domains dealing with chemical exposure develop slowly. This widens the gap between the scientific state-of-the-art and use in regulatory frameworks [33]. Risk managers and policy makers therefore face decision making with often outdated, incomplete or inconsistent exposure information, thus hampering an efficient, effective and consistent management of risks [47].

Over the last 4 years, the ‘Europe Regional Chapter of the International Society of Exposure Science’ (ISES Europe) mobilised experts from different disciplines, policy domains and stakeholder groups to jointly prepare the foundation for a European Strategy on Exposure Science 2020–2030 [21, 48]. As part of this strategy, the goal of the present paper is to identify requirements and provide a way forward for aligning and strengthening the cross-policy uptake and application of exposure information in current and future EU policies. To achieve this goal, the ISES Europe ‘Working Group on Integrated Framework of Exposure Science and Policy Efficiency’ pursued three specific objectives: (1) to provide an overview of the current use of exposure information across major EU policy domains; (2) to identify where methodologies using the same exposure information can be aligned across EU policy domains, in support of regulatory harmonisation of exposure information; and (3) to propose the frame for the development of baseline acceptance criteria for exposure data, methods and tools, and aligned use of exposure information across current and future EU legislation, supporting a ‘One Substance – One Assessment’ approach.

## Mapping of EU Legislation with provisions on exposure information

**Fig. 1:**
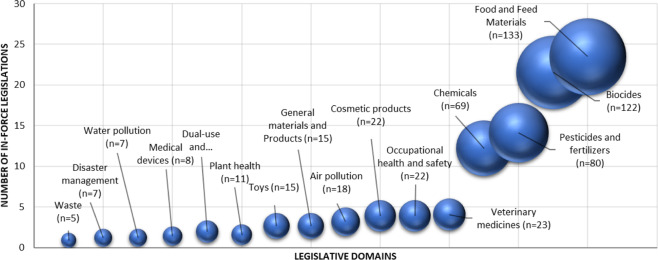
EU chemical management legislation clustered into 16 domains making use of exposure information. The size reflects the number of in-force legal instruments comprising Commission Regulations, Commission Directives, Commission Implementing Regulations, Council Regulations, Council Directives, Directives of the European Parliament and of the Council and Regulations of the European Parliament and of the Council.

To create a snapshot of the current EU regulatory landscape with provisions making use of exposure information, search queries were made in EURLEX (https://eur-lex.europa.eu/homepage.html) using combinations of ‘exposure’ with a topical term covering the domain of chemicals (i.e., biocid*, chemical*, substance*, pesticid*, plant protection*, pharmaceutic*, medic*, cosmetic*, pollutant* and contamina*) resulting in 566 records with full-text regulations and directives of in-force legislation. Records were clustered into 16 policy domains relevant for chemicals management that make use of exposure information domains with varying numbers of records. To account for sub-regulations within a certain domain, the number of records within each domain was divided by the total number of records for all domains, thus creating a weighted domain size as illustrated in Fig. 1.

Figure 1 shows that exposure science is associated with all chemicals-related policy areas including biocides, food and feed, chemical- and product-level legislation, environmental media, occupational health, disaster management, dual-use and defence and waste. Consumer product legislation is shown for some specific categories (e.g., toys, cosmetics) or grouped under ‘general materials and products’. On the far-right side of Fig. 1, the domain of ‘food and feed’ is the largest in terms of number of legal instruments (*n* = 133) where exposure is relevant. The numbers are determined by the legal architecture of the decision-making processes in a certain policy area and reflect the regulatory density in the different domains.

**Fig. 2:**
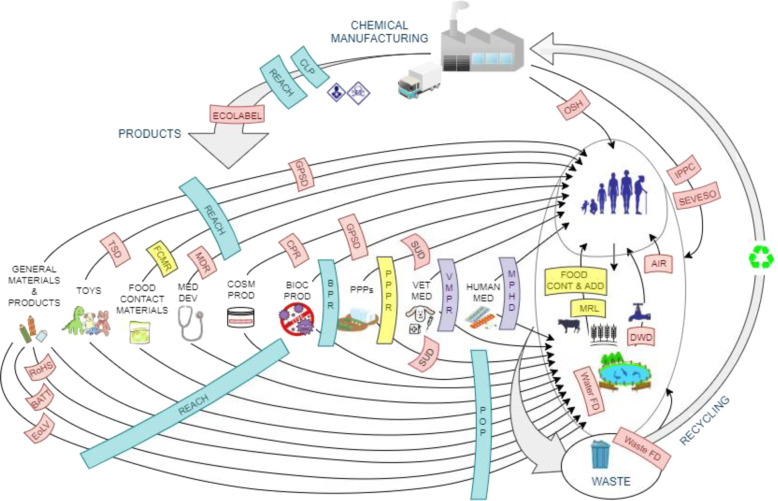
EU legislation with chemical risk management provisions mapped against envisaged pathways (black arrows) of human and environmental exposure. Legislation falling under the scientific remit of different EU institutions is labelled with different colours (blue: ECHA, yellow: EFSA, purple: EMA, red: EU Commission and Scientific Committees). CLP Classification, Labelling and Packaging of substances and mixtures, regulation (EC) No 1272/2008, REACH Registration, Evaluation, Authorisation and Restriction of Chemicals, regulation (EC) No 1907/2006, ECOLABEL EU Ecolabel, regulation (EC) No 66/2010, GPSD General Product Safety Directive 2001/95/EC, TDS Toy Safety Directive 2009/48/EC, FCMR Food Contact Materials, regulation (EC) No 1935/2004, MDR Medical Devices, regulation (EU) 2017/745, CPR Cosmetic Products, regulation (EC) No 1223/2009, BPR Biocidal Products, regulation (EU) No 528/2012, PPPR Plant Protection Products, regulation (EC) No 1107/2009, SUD Sustainable Use of Pesticides Directive 2009/128/EC, VMPR Veterinary Medicinal Products, regulation (EU) 2019/6, MPHD Medicinal Products for Human Use Directive 2001/83/EC, RoHS Restriction of Hazardous Substances in Electric and Electronic Equipment, Directive 2011/65/EU, BATT Battery Directive 2006/66/EC, EoLV End of Life Vehicles Directive 2000/53/EC, POP Persistent Organic Pollutants, regulation (EU) 2019/1021, Water FD Water Framework directive 2000/60/EC, Waste FD Waste Framework directive 2008/98/EC, DWD Drinking Water Directive 98/83/EC, MRL Maximum Residue Levels of Pesticides, regulation (EC) No 396/2005; FOOD CONT & ADD Food Contaminant regulations (EEC) No 315/93 and Food Additives regulation (EC) No 1333/2008, AIR Ambient Air Quality and Cleaner Air for Europe directive 2008/50/EC, SEVESO Seveso III directive 2012/18/EU, IPPC Integrated Pollution Prevention and Control directive 2010/75/EU, OSH Occupational Safety and Health Legislation, including directives 98/24/EC, 2004/37/EC, 92/85/EEC 94/33/EC.

Another way to analyse how exposure science feeds into scientific assessments across policy domains is to map EU legislation regulating chemicals against pathways of human and environmental exposure (Fig. 2). Legislation can trigger interventions to mitigate exposure arising at various stages of material and product life-cycles; several product-level regulations and directives are closely interconnected with horizontal legislation (e.g., REACH). The same product type (e.g., cosmetic products, food and feed) may be covered by different legislation with respect to relevant human and environmental exposure pathways. Figure 2 further illustrates that while risk management may be sector-specific, exposure and risks to humans and the environment may result from multiple sources and exposure pathways, thus being relevant across sectors. A comprehensive science and policy framework facilitating use and exchangeability of exposure information and assessments is hence essential to efficiently capture overall risks.

The sectorial nature of the current framework, and especially of product-level legislation, is partly motivated by the choice of the legislator to tailor risk management to policy-specific objectives and constraints. Accordingly, guidance and tools for exposure assessment have evolved sector-specifically, designed to meet the needs of specific policy domains. With that, the current policy framework appears fragmented rather than integrated. It also reflects a linear economy model, falling short of capturing multiple circularity processes (UNEP, [24]).

**Fig. 3:**
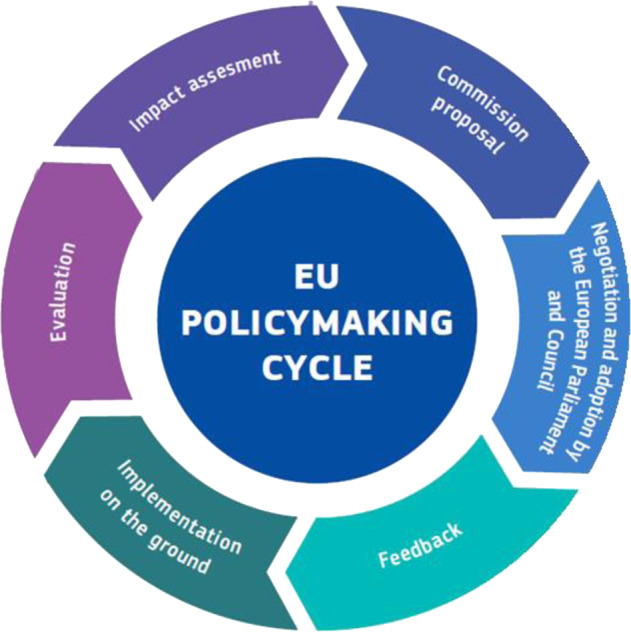
The EU Policy Framework. The full policy cycle comprises the stages of proposing, adoption, implementation, evaluation and revision. Exposure information is key to policy implementation, evaluation, and impact assessment of policy options.

Another dimension to consider when mapping exposure science inputs to European legislation is the dynamic nature of the policy cycle. The full cycle (Fig. 3, adapted from and according to the EU Policymaking Hub (https://knowledge4policy.ec.europa.eu)) comprises policy design with impact assessment of policy options, adoption, implementation, application (enforcement and monitoring), evaluation and revision. At each phase, the Commission is guided by its policy design principles [49] to make sure that the EU strives for continuous improvement in identifying and addressing policy needs through objective evaluations and stakeholder consultations. Exposure science supports all the inter-related phases of the policy cycle of legislation that involve global exposure information and especially the implementation, evaluation and impact assessment of policy options.

## Exposure science challenges to strengthen policy frameworks

Building on the findings of recent EU policy evaluations and the outcome of three ISES Europe workshops held in 2018 (Federal Institute for Occupational Safety and Health, Dortmund, Germany), 2019 (RIVM, Dutch Institute for Public Health and the Environment, Bilthoven, The Netherlands) and 2020 (European Commission’s Joint Research Centre, Ispra, Italy), the working group on ‘Integrated Framework of Exposure Science and Policy Efficiency’ identified seven challenges that need to be addressed to strengthen the relevant legislation by contribution of exposure science across EU policy frameworks:

1. Availability of exposure data, information and knowledge for use across policy domains.
2. Acceptance criteria for exposure data and methods across policies.
3. Integration of scientific exposure assessment and modelling frameworks.
4. Integration of exposure knowledge into companies’ management systems.
5. Regulatory adoption of innovative monitoring approaches.
6. Consideration of combined exposure to multiple chemicals.
7. Harmonising the use of exposure science across all relevant policy domains.

### Availability of exposure data, information and knowledge for use across policy domains

The availability of reliable use and exposure information plays a key role across several policy areas (see Figs. 2 and 3). Some policy areas still lack basic exposure information requirements to enable successful implementation. For example, pesticide use data collected under the Pesticides Statistics Regulation (EC) 1185/2009 [50] and made available in EUROSTAT are so heterogeneous that they cannot be used to draw reliable conclusions on pesticides use and emissions in the EU [51, 52]. Incomplete data on pesticide use at the EU level currently hinders progress towards the objectives of Directive 2009/128/EC on the Sustainable Use of Pesticides [53, 54]. Heterogeneous pesticides data also represent the main source of uncertainty of EU-scale model estimations of environmental exposure to pesticides, which was meant to be useful to inform risk managers under the Water Framework Directive [51]. Harmonisation and aggregation of exposure data at the EU level is a recurrent challenge across policies. Another illustration for the lack of accessible information on the uses of chemicals is the per- and polyfluoroalkyl substances (PFAS) case (e.g., as used in numerous consumer products) [55]. A huge variety of substances belonging to the PFAS family can be detected in the environment, and—despite registration requirements under REACH—there are very few data available based on which it would be possible to track back to the source of release. Aggregated data for assessing occupational risks from chemical exposure only exists at the national level in some Member States, and is not collected in a harmonised way at the EU level, preventing their potential use for other policies, such as REACH. Vice versa when available, REACH tools capturing occupational exposure scenarios are not used to inform Occupational Safety and Health legislation [34]. While there is no specific legal requirement on all employers requiring the mandatory provision of information on the exposed working population [56], employers are obliged to report on carcinogens and mutagens uses and exposure under Directive 2004/37/EC when specifically requested by the national authorities [57]. The lack of such information currently hinders the implementation of targeted risk management measures.

In most cases, exposure information requirements have been designed for individual pieces of legislation. Therefore, it is key to establish mechanisms that allow the multiple use of chemical and other relevant data beyond a specific regulatory domain. Additional obstacles related to the structure, quality and accessibility of data, e.g., REACH, currently limit the potential for broader use to tackle policy and scientific questions [21]. Promoting the harmonisation of data reporting standards irrespective of legislative domains improves interoperability and reduces costs and the need for unnecessary testing [58]. It is important to reduce barriers of data sharing and exchange across policy domains moving towards open data policies, while conforming to the requirements of the General Data Protection Regulation (GDPR), for example, by anonymization of sensitive personal data [59].

### Acceptance criteria for data and methods across policies

Criteria for reliability and acceptance of exposure data and tools vary across EU legislation. Different standardisation and acceptance approaches have been followed at the EU or international level. Most exposure assessment approaches are, however, not subject to international mutual acceptance programmes, such as the OECD mutual acceptance of data [60]. In some cases, modelling and monitoring approaches follow prescriptive protocols, tools (e.g., models) and governance mechanisms at the EU level. One well-known example is the EUSES software [61–66], containing a wealth of exposure data and algorithms to carry out assessments for industrial chemicals and biocides. However, because it is a harmonised tool with high international acceptance, reflecting scientific progress through regular updates is a slow and difficult process. Concerning monitoring data, despite standard guidance and protocols being in place, such as for monitoring under the European Water Framework Directive [6], inconsistencies occur related to their poor implementation (e.g., missing reporting of limit of detection/quantification). In addition, there is a lack of analytical standards to quantify detected exposure, and there is a lack of data and/or methods for traceability [3]. In other cases, data and assessments are accepted based on a more flexible case-by-case evaluation. For human occupational exposure modelling, for example, various competing tools exist. Depending on the tool, exposure predictions for the same use-case scenario can differ significantly and are frequently not in line with measured datasets [67–69]. Exposure assessments are hence frequently inconsistent across sectors and sometimes even among registrants of the same chemical substance [70].

A specific case concerns physiologically based kinetic models, which are playing an increasing role in the estimation of internal exposure, among others in conjunction with in vitro-to-in vivo extrapolation in emerging alternative methods to animal testing (e.g., [71]). In this case, despite guidance and standardisation efforts achieved at the OECD level [72], regulatory authorities still face the challenge of developing new scientific capability to embed new model-based solutions as part of integrated approaches to testing and assessment that deviate from conventional paradigms [73].

The rationale behind different expectations and quality acceptance criteria used under different EU legislation is perceived as incoherent by the scientific community, and thus could undermine science-based legislative decisions. This apparent incoherence is partly explained by the lack of baseline acceptance criteria for exposure information, estimation methods and models across policy domains. While progress has been made to make exposure information from different sources available, the regulatory adoption of these initiatives depends on the acceptance of data across policy areas. The European Commission’s Information Platform for Chemical Monitoring (IPCHEM, https://ipchem.jrc.ec.europa.eu/) [74, 75] and the Network of reference laboratories, research centres and related organisations for monitoring of emerging environmental substances (NORMAN network) [76], for example, have achieved a wide recognition and acceptance over the years in the EU and at the international level, as they collate and make chemical monitoring and other data from various regulatory and research contexts available [77]. The success of shared data platform and exposure assessment tools requires shared quality control rules throughout the data life-cycle [78]. This can only be achieved through an unprecedented level of commitment and collaboration among regulatory authorities and other stakeholders towards a common and widely adopted scientific framework for exposure assessment.

### Integration of scientific exposure assessment frameworks

The existing risk assessment frameworks fall short of integrating various exposure information across all relevant sectors. That is, information related to the field of exposure science, defined as the contact between stressors and receptors, and the associated exposure sources, exposure pathways and processes potentially leading to impacts on human health and the natural and built environment [79] (see Fig. 2).

Assessment tools, methodologies as well as ontologies (e.g., use categories) have been developed to meet the needs of particular legislations. This has led to inefficiencies in assessment processes that need to integrate exposure knowledge from various EU policy domains. In some cases, this situation has led to differences in regulatory action when the same (group of) substances have been assessed under different legislation. This was, for example, the case for the recent interventions on phthalates under REACH and the Food Contact Material Regulation (see Box 1).

Situations, such as the one described in Box 1, point to the need of establishing a common framework enabling integrated exposure assessments, tracking chemicals of concern from sources to relevant receptors via all potential exposure pathways. Past EU-funded projects developing integrated exposure assessment tools (e.g., EIS-ChemRisks Toolbox [80–82] led to limited regulatory uptake and use. These experiences indicate that commitment and co-design of solutions by policy makers and exposure scientists are prerequisites for success. EU legislators should furthermore take advantage of new data, concepts and assessment tools, such as the adverse outcome pathways (AOP) concept, to optimise the adoption of existing and new exposure information. It has been proposed that, for example, the combination of aggregate exposure pathways with AOPs optimises the use of existing exposure data, for example, by developing scenario relevant dosing, and enabling in vitro-in vivo extrapolations [83]. A harmonised scientific framework is, however, only part of the solution. Improved policy coordination is also needed. To this end, the risk management option analysis developed under REACH promotes common understanding and early discussions towards appropriate interventions under various pieces of legislation. Its implementation by ECHA has proved to be an effective process, allowing information sharing among authorities and stakeholders across policies [35].

Box 1 Interventions on phthalates under REACH and the Food Contact Material Regulation Low molecular weight phthalates were assessed for their risk to human health under REACH by ECHA in 2017, and subsequently by EFSA in 2019, specifically for concerns related to their use in food contact materials [115]. The two assessments were carried out largely independently, without alignment with respect to the definition of chemical grouping, in the exposure scenarios considered and, consequently, in the methods and exposure data used (e.g., population age grouping, food intake estimates).

By using HBM data for the exposure assessment, ECHA pointed to evidence of risk to human health from combined exposure to four phthalates and of a large contribution of exposure to di(2-ethylhexyl) phthalate from the diet based on modelling estimates [116]. According to the mandate received, EFSA’s assessment focussed on dietary exposure from phthalates authorised for use in food contact materials. The conclusion was that dietary exposure did not result in exceeding group-based or individual tolerable daily intake. It is obviously more likely to conclude on the exceedance of a given threshold when considering aggregate exposure across possible sources, as compared to only considering a single exposure source. The two assessments are consistent within the mandate received, but the rationale for different regulatory intervention is difficult to justify from a cross-policy standpoint.

### Integration of exposure knowledge into companies’ chemical management systems

Companies being part of the chemicals supply chains need to have management systems in place for, e.g., (i) preventing accidents at major production or storage sites or during transport, and (ii) ensuring that chemical substances in mixtures, materials and articles can be used in a safe and sustainable manner. Traditionally, such management systems target production or manufacturing site-related aspects as well as hazard classification of chemicals, whereas development of product safety management and safe-and-sustainable product design are more recent trends.

In 2006, REACH has introduced the obligation for manufacturers and importers of chemical substances to register their substances, to assess the hazards based on obligatory tests and to map the uses of each substance over its entire life-cycle. For hazardous substances, a chemical safety assessment (CSA) must be carried out for the substance as such, the substance in mixtures and substance in articles. The CSA method is prescribed in Annex I to REACH. This requires REACH registrants to collect information on uses, conditions of use and exposure from their supply chains, to describe and quantify the releases from their products accordingly, in order to be able to demonstrate safe use of such substances. Where not possible (based on existing conditions), registrants need to work out the additional risk management measures. At the end, the conditions of safe use (final exposure scenarios) must be communicated down the supply chain via safety data sheets. Downstream users must verify that the uses indeed take place under the conditions assumed by the registrant, and otherwise adapt or carry out their own assessment. However, the REACH paradigm of CSA across the supply chain also introduced new challenges for exposure science, including:

- The REACH CSA takes the product safety perspective and therefore requires collection of use and exposure knowledge from many players along the supply chain. Such type of exposure assessment differs from the classical single-site assessment to protect workers and environments. It requires the analysis of use patterns and volume tracking (mass-flow analysis) to be connected with the more classical ways of measuring or modelling exposure at a single site or workplace. Some trade and industry sector organisations have started to use ‘collective’ market knowledge for generating information required for generic exposure assessment, which is currently structured in the form of sectorial use-maps libraries. Whether or not such voluntary initiatives provide sufficient information to enable generic exposure assessment by manufacturers is still to be seen, no systematic evaluation yet exists. The needs and approaches for further developing robust exposure assessment tools for substances manufacturers are discussed in Schlüter et al. [84].
- To enable communication of verifiable risk management advice down the supply chain to the users of chemicals (including producers of mixtures and articles), exposure assessment parameters need to be ‘translated’ into risk management advice [85]. Such advice must be understood by industrial hygienists, environmental managers, product developers and product safety managers. Small companies, which make the majority of chemicals users, should receive the advice in a readily applicable and understandable form. ECHA along with the owners of various exposure tools applicable under REACH made a first attempt to ‘harmonise’ the way in which the conditions of use driving exposure are expressed [86]. This has been fed into a multi-annual work programme for improving the efficiency and effectiveness of communication about exposure and risk management up and down the supply chain, developed by ECHA, industry and Member States under the Commission’s last REACH review [87]. This includes, for example, a harmonised structured format for the transfer of safety data through the supply chain.
- REACH is based on the principle of ‘One Substance – One Registration’, aiming to make all companies placing the same substance on the market to come up with one common data-set in harmonised electronic format (IUCLID), characterising the properties and hazards of the substance. However, there is no requirement yet to conduct and maintain one common safety assessment per substance, and hence suppliers provide diverse and partly conflicting risk management advice for the same substance under the same conditions of use.

Improving the management of use and exposure information within and across the supply chains of chemicals will require changes in companies’ management systems, improved methods to predict the use and the behaviour of hazardous substances under use conditions, digitalised data transfer and better co-operation mechanisms within and across industry sectors. Improving the access of authorities (and general public) to up-to-date, quantitative information about production and uses (including processing into articles) of chemicals may even require better legal mechanisms than the current REACH system, in order to retrieve the distributed knowledge about uses from the market actors, and at the same time ensuring data protection and competition law.

### Regulatory adoption of innovative monitoring approaches

The ISES Europe community of exposure scientists has concluded that the gap between the scientific state-of-the-art and regulatory implementation has widened in recent years [32, 33]. Scientific progress in human and environmental monitoring has the potential to improve the representativeness and the biological relevance of monitoring information. Non-target chemical analysis, as well as exposure and effect biomarkers can broaden the scope of current target monitoring to better address environmental mixtures for human and environmental exposure assessment [88]. Passive sampling provides time-integrated measurements concentrations in aqueous and gaseous phases, better reflecting substances bioavailability. HBM of chemicals and their metabolites provides exposure information integrating over multiple sources and pathways while accounting for the toxicokinetics that govern internal exposure [89, 90]. All these methods are not fully exploited yet (see Box 2).

The implementation of new scientific approaches in regulatory provisions and processes requires commitments from all actors. New incentive mechanisms need to be explored to motivate scientists to take up the challenge and the associated resource implications. There are, however, certain steps in the policy cycle (Fig. 3: policy ‘evaluation’ and ‘impact assessment’ of different policy options) where new science may be deployed without the constraints of full regulatory implementation [19].

Box 2 Challenges for the regulatory uptake of human biomonitoring (HBM) data The case of HBM exemplifies how regulatory adoption can be hampered due to a combination of technical (e.g., reproducibility, standardisation), legal (data protection and privacy policy) and policy-related issues (e.g., relevance to risk managers, fitness of legal provisions). Legislation often requires the consideration of all scientific evidence in carrying out assessments, including data from academic (non-regulatory) studies. This was the case for recent assessments done for phthalates and bisphenol-A restrictions and 4,4’-methylenebis[2-chloroaniline] authorisation under REACH [45]. However, the absence of regulatory requirements as well as the lack of standardisation of sampling design and analytical procedures has slowed regulatory adoption. Sharing biomonitoring data among scientists and regulators also faces legal constraints, such as those established under the General Data Protection Regulation (EU) 2016/679 (GDPR) [59]. Data anonymization and the use of new technologies for reliable encryption of data, such as blockchain, are some of the possible alternatives to unlock the HBM data and to improve its scientific value, while ensuring compliance with data protection policies. The case of HBM points to the need of establishing an early dialogue between scientific and policy stakeholders to reach a common understanding about the nature (technical, legal or policy-related) of the challenge(s) that stand in the way between scientific progress and regulatory adoption, and to jointly identify mutually acceptable solutions.

From the perspective of risk managers, one important limitation of HBM is the difficulty in tracing the internal exposure back to the sources, which in principle might hamper effective risk management measures. Reconstructing the sources and exposure pathways driving human exposure represents a huge challenge given the multitude of combinations of chemicals, products, and exposure pathways, which can be achieved through iterative processes of integrating HBM data with mechanistic models. However, a systematic solution requires an integrated model framework, which is currently missing.

### Consideration of combined exposure to multiple chemicals

Examples have shown that chemicals, individually present at levels that do not adversely affect human health or the environment, may cause harm when they occur in combination with each other [10, 27]. Several reviews and case studies have addressed this issue by analysing current regulatory requirements [91], scientific methods, challenges and possible ways forward [10]. To date, regulatory provisions and related guidance exist in several EU legislative areas to assess and manage intentional/commercial mixtures of chemicals. In contrast, unintentional mixtures (i.e., exposure of ecosystems or humans to multiple chemicals from various sources) are rarely considered, with few exceptions including pesticide residues (MRLs), some examples of grouping assessments and management processes, mainly under REACH (e.g., phthalates, PFAS [10]), and the proposed implementation of bioassays for mixture effects (e.g., effect-based methods) for water quality assessments under the Water Framework Directive [92].

Despite recent scientific progress, regulatory adoption is still slow. Partly, this is due to technical issues, such as the lack of standardised methodologies, particularly regarding the selection of substance combinations to prioritise from a virtually infinite combination of exposures [40]. Perhaps most importantly, limited regulatory adoption is due to the complications that mixture assessments bring to risk management. When a risk is identified for a given combined exposure, the question remains about which sectors and which chemicals should be regulated/restricted [10]. Risk assessment results from combined exposure to multiple chemicals need to be followed by substance- and sector-specific interventions based on socio-economic considerations, such as the benefits of specific uses and the availability of alternatives. In the short-medium term, possible solutions include the combined assessment of groups of substances (based on similar functionality or toxic mode(s) of action) or the use of mixture assessment factors (an extra safety factor in risk characterisation that accounts for unknown combination of chemical exposure) [93].

In the longer term, the assessment of (unintended) environmental mixtures should be fully explored in the broader policy cycle. Recent related evaluations of EU policy could not take advantage of state-of-the-art methodologies to evaluate the overall effectiveness of chemical legislation. Evidence was limited to exposure and risk of a few single substances (e.g., lead and lead-related disease incidence) [34]. The next cycle of policy evaluation would benefit from the deployment of component-based assessments (e.g., additivity-based mixture risk indicators) and whole-mixture approaches (e.g., effect-based methods) to monitor the progress towards new policy objectives (e.g., 50% reduction in use and risk from pesticides [1, 94, 95]). In the EU Chemicals Strategy for Sustainability, the European Commission committed to assess how best to introduce mixture assessment factor(s) in Annex I of REACH for the CSA of combined exposure to non-intentional mixtures of chemicals [18].

### Harmonising the use of exposure science across all relevant policy domains

Protection of human and environmental health against threats is a common objective across all chemical-related policies. Administrations with limited knowledge and capacity to manage chemicals present a risk to health and the environment but also a security threat. Intended chemical poisoning incidences using consumer products, food items and chemical weapons are examples of security threats [12, 96, 97]. A possible down-side of increased exposure knowledge might be that prevention of intended incidences becomes increasingly challenging. Knowledge control mechanisms are needed to avoid misuse of readily available and accessible information on the production use, and delivery of chemicals that may have a dual-use (see Box 3).

Existing chemical assessments are often focused on managing the risks posed by chemicals to human health, including occupational health and safety and environmental health. However, considered risks posed are often related to intended chemical use, accidental or negligent misuse of chemicals, while the scope of the assessments do currently not consider that risks may also be related to the intentional misuse of chemicals [98]. Professionals dealing with chemicals therefore should be as aware of chemical security issues as of chemical safety issues [12]. Compared to chemical safety, chemical security policy deals with risks characterised by lower likelihood but higher potential consequences. Despite the different sources of uncertainty, generally the same scientific principles apply for hazard and risk assessment, while accounting for likely differences in exposure settings (e.g., magnitude of exposure). The two policy domains, however, are only loosely interconnected. Specifically, EU policies dealing with chemicals with a direct or indirect focus on security risks (e.g., the Dual-use Regulation and the Seveso Directive) would benefit from improved use of available exposure and risk knowledge. Furthermore, better alignment of scientific approaches used under chemical safety legislation would be advantageous.

Globally, knowledge about the identification of chemicals on the market, their properties and their uses remains scattered [3, 23]. The lack of harmonised global chemical inventories is a major obstacle to prioritise and monitor global safety, security and sustainability issues. Limited implementation of sound chemicals management principles defined under international agreements (SAICM) jeopardises the enforcement of international treaties regulating the trade of hazardous chemicals (Rotterdam Convention) and waste (Basel Convention). The existence of loopholes regarding international chemicals trade adds to the security risks as acknowledged by organisations, such as the International Marine Organization, the International Civil Aviation Organization, UN Environment, the UN Food and Agriculture Organization and the WHO [23]. For example, the provisions of the Rotterdam Convention only apply to shipments falling under the use categories mentioned in the Annex III listing (pesticides and industrial, http://www.pic.int/TheConvention/Chemicals/AnnexIIIChemicals). This allows exporters to claim an ‘incorrect’ use category to bypass rules, deliberately causing a potential risk.

Security-driven risk assessment needs to estimate the likelihood that hazardous substances are intentionally used to cause harm and the potential of eventual consequences of related incidents. Owing to the difficulty to establish the criteria against which risk should be assessed, there is hardly any guidance defining the scope of assessments or methodologies to systematically address this problem. Consequence-assessment models estimate health and environmental impacts based on toxic release and dispersion algorithms in potential combination with thermal radiation of chemical fires and explosions of vapour flammable clouds [99], and on vulnerability assessments. They have been developed in the context of the EU Seveso III Directive for accidents but are also used in the security context [100]. In this case, however, their scope is typically limited to short-term exposure scenarios and associated impacts. Similarly, for security risk assessment of dual-use substances (both civilian and military purposes), international frameworks have not provided specific guidance to direct individual human risk assessment [101]. Any national authority responsible for granting a licence considers the nature of the goods, the country of destination, the end-user and the proposed end use [102]. Apart from the export control list, however, ‘there is hardly any guidance’ for the assessment of the risk of misuses [101]. The consequences of ill-informed assessments can be disastrous.

International actors need to step up efforts to build a shared knowledge base and technical capacity to better identify and assess security threats and to improve coordination of risk management (prevention and preparedness). Interconnecting exposure science across health, safety and security regulatory domains comprise (i) chemical inventories and tools facilitating tracking of hazardous substances, materials and waste, (ii) exposure and risk assessment models for intended or unintended release (accident, fate and exposure models, but also foresight methods) and (iii) chemical monitoring including timely environmental and human (bio)monitoring following emergencies.

Box 3 An example of malicious trade and misuse of chemicals Council Regulation (EC) No 428/2009 deals with the export of dual-use items, including substances used for both civilian and military purposes. Its infringement impacts both safety and security risks especially when it concerns a chemical substance with a dual-use (both civilian and military purposes) such as listed in Annex I of the Regulation. For example, sodium fluoride used in the fluoridation of drinking water and the production of toothpaste and phosphorus trichloride used in the production of organophosphate insecticides and glyphosate herbicides are two cases where misuse occurred. Sodium fluoride and phosphorus trichloride were employed to synthesise the deadly chemical weapon, sarin. In 2013, a UN investigation found clear and convincing evidence that sarin was used against civilians in the Ghouta area of Damascus, Syria on 21 August 2013 and promptly after the incident, blame was directed in part at the exporters of chemical compounds from the UK, and Germany [101].

## Priority areas and recommendations

The present paper focuses on the specific requirements and actions relevant to increase the use exposure science and its regulatory uptake in Europe. To address the challenges outlined above, also with a view to their global dimension, we have identified five key areas for actions:

1. Creating a common scientific framework for exposure assessment interfacing EU chemical policies for environment, health, safety/risk and sustainability assessments, with particular emphasis on different exposure aspects, including common terminology, common principles and a suite of accepted data, methods and tools. EU Agencies are well placed to coordinate the development of an overarching scientific guidance describing how exposure data, models and knowledge fit together, considering terminologies, principles, methods and tools inside and outside EU. The main principles and core method described in REACH Annex I for CSA may provide a suitable starting point. Scientists and policy makers could jointly explore how existing scientific concepts and tools for integrated human and environmental exposure assessment could contribute to a comprehensive, modular scientific framework. Member States’ Competent Authorities, industry and the scientific community should contribute to identify opportunities for linking and consolidating approaches with the ambition to establish internationally recognised standards and tools (e.g., IPCHEM for monitoring data and the NORMAN network for database systems), supporting and facilitating data sharing, harmonisation and coordinated research and development globally [76, 78, 103, 104]. Implementing common standards in documenting metadata (e.g., building on the existing IUCLID formatting for use and exposure data) stimulates data quality and acceptance criteria, streamlining data generation, storage and processing. Data owners should raise their commitments to reduce barriers to open sharing of data, information and knowledge. In line with the intention to simplify and strengthen the legal framework, EU institutions should embrace a holistic, cross-policy mindset, where risks are identified based on knowledge on overall exposure; and coordinated risk management measures shall be based on facilitating sharing knowledge of all relevant uses, emission sources and exposure pathways and settings along entire chemical and product life-cycles. Testing the validity and usefulness of the aforementioned framework via use cases requiring cross-sectorial assessment and management would improve the overall EU chemicals policy and regulation fitness.
2. Improving the coordination of regulatory processes. A common scientific framework can facilitate risk assessors to coordinate their assessments. In addition, also requires strong commitment by policy actors to implement the mechanisms for policy integration and coordination. This includes improved coordination of assessments and management processes in horizontal chemicals legislation (e.g., REACH), product-level legislation and downstream legislation (e.g., industrial emissions, occupational health and safety, waste) and environmental quality control (water/air legislation). This has been recognised by the Commission in its commitment to implement the principle of ‘One Substance – One Assessment’ [7]. Its success requires strong commitment from all policy actors to facilitate the exchange of exposure data and knowledge, and to coordinate risk management processes (e.g., through analyses of risk management options).
3. Integration of exposure knowledge into companies’ management systems is a prerequisite to ensure safety and sustainability of products and processes, and hence to securing (global) business. Responsibility put on industry is a paradigm commonly encountered in several pieces of chemical legislation; however, practical implementation is still lacking. Firstly, chemicals health and safety risk evaluation and management expertise and knowledge should be integrated across business functions (e.g., R&D, product design, supply chain, legal affairs, engagement of the public) rather than being addressed as only a compliance task that can eventually even be outsourced [105]. Designing safe and sustainable chemical-based processes, mixtures or articles requires application and further development of methods to predict the behaviour of hazardous substances under conditions of use and under conditions of a more circular economy, i.e., through recycling, repurposing, remanufacturing, etc. Secondly, for manufactured hazardous substances as such (and/or its constituents and impurities), and for mixtures, materials (virgin and recycled) or final articles made from them, the supplier should provide structured and harmonised safety datasets. Such datasets should include information on concentration of hazardous ingredients, the ingredients’ function and intrinsic properties, the hazard and exposure characteristic, the extent of knowledge available on the non-hazardous ingredients and the conditions of safe and sustainable use (exposure scenarios). The legislative framework for obliging producers to provide such data exists; however, implementation and enforcement is lacking. Thirdly, the flow of safety data along supply chains (i.e., between companies) needs to be improved via digitalisation. Industries’ readiness to invest in a harmonised electronic system for exchange of safety data in the market may need some regulatory support (e.g., provision of electronic datasets as an obligation). Finally, more knowledge and resources are needed in industry sector organisations, to provide companies with support in organising efficient and effective communication on chemicals safety and sustainability along and across supply chains.
4. Improving the uptake of exposure science innovation into the policy cycle. Scientists and policy makers should take advantage of existing mechanisms (e.g., stakeholder consultations and scientific input to policy evaluations and impact assessments of existing and new policies), to ensure that policy developments benefit from innovations in exposure science. EU-funded research should better respond to the gaps in knowledge identified through policy evaluations (e.g., lack of methods to assess the behaviour of hazardous chemicals in materials, including recycling materials). Following the adoption of the Chemicals Strategy for Sustainability [7], new mechanisms are being explored to engage scientists in the analysis of how exposure science innovations can contribute to policy needs (e.g., Partnership for the Assessment of Risk from Chemicals) [106]. Scientists and policy makers should establish a stronger dialogue from the onset of research projects to identify the optimal entry point of new science into the policy cycle and ensure its broad acceptance across all stakeholders concerned, e.g., as seen in recent global consensus-building efforts under UN Environment [107]. Policy uptake of science is not limited to the implementation of legislation. For example, HBM, mixture assessment approaches, biomarkers of exposure and effects can be useful not only to support regulatory risk assessments but also to monitor progress towards achieving policy objectives [89]. They should be deployed now to inform the next cycle of policy evaluations. New modelling approaches can be used in prospective studies supporting the impact assessment of policy options, which precede new policy proposals.
5. Harmonising and utilising exposure science across health, safety and security policies. At the international level, improved access to global chemical inventories, exposure data, tools, guidance and knowledge enables stronger connections across scientific frameworks underpinning chemical health, safety and security policies [23]. National authorities should join forces to create a global inventory of chemicals on marketed compounds shared among all parties in research and regulation to support screening and monitoring of hazardous chemicals [3, 76, 108–110]. For regions with developed policies, such as the EU, this provides insights on chemical safety and security threats that may enter their jurisdictions through global trade flows. Building on globally recognised inventories, international co-operation should aim at establishing processes and tools to track and monitor flows of hazardous chemicals from the various sources and with various destinations across countries: e.g., resource extraction, agriculture, industrial manufacturing and energy generation, manufactured materials, products (including expired products, such as pesticides and medicines), waste and environmental media within [47, 110, 111]. National stakeholders should be made accountable to maintain and share such information according to international agreements (e.g., SAICM, Rotterdam Convention).

At the EU level, chemical hazards and risks identified under policies dealing with direct and indirect security matters (e.g., Directive 2008/68/EC on the inland transport of dangerous goods, Directive 2012/18/EU on the control of major-accident hazards involving dangerous substances, or Council Regulation (EC) No 428/2009 setting up a Community regime for the control of exports, transfer, brokering and transit of dual-use items) should be assessed and managed based on the same scientific principles used under chemical safety legislation. For example, risk assessments triggered for dual-use substances and major-accident risks involving dangerous substances usually focus on the short-term consequences of potential exposures following accidents (unintentional release) or incidences (intentional release). Their scope should include the potential effects on human health of long-term exposures, in line with scientific guidance established for unintentional chemical accidents. Assessments should take advantage of data and tools available under chemical legislation (e.g., REACH). For certain identified hazards (e.g., flammability, inhalation toxicity) and security risks (e.g., dual-use chemicals), coordinated solutions to risk communication and management should be considered [85, 112–114]. One option, for example, is the development of chemical security classification and labelling for use by government officials working on export licensing and chemical traders. Another example concerns the need to optimise risk management responses to accidents or to identify security risks. Following emergency situations, regulatory triggers and guideline protocols for monitoring (e.g., HBM, environmental monitoring) could be aligned between civil protection policies covering both accidents (e.g., Seveso directive) and intended events (e.g., dual-use regulation).

## The next steps towards 2030

The present ‘European Exposure Science Strategy 2020–2030’ is the starting point aimed at engaging all relevant European and global stakeholders to ensuring that its implementation will lead to an ultimately more efficient development, implementation and acceptance of exposure science and its underlying data, information and knowledge and related application across EU and global policy domains. All relevant stakeholders concerned are encouraged to align their strategic institutional and organisational objectives and targets embedding exposure science into current and future operating modes within a synergistic context and perspective. In the light of the absence of a thorough global analysis, both the process and outcome of the European exposure science strategy would be helpful for other geographical areas around the world boosting global policy uptake of exposure science embedded into global policy strategies and frameworks (e.g., the UN SDG programme).
